# The triple threat of pregnancy, HIV infection and malaria: reported causes of maternal mortality in two nationwide health facility assessments in Mozambique, 2007 and 2012

**DOI:** 10.1186/s12884-015-0725-7

**Published:** 2015-11-09

**Authors:** Patricia E. Bailey, Emily Keyes, Allisyn C. Moran, Kavita Singh, Leonardo Chavane, Baltazar Chilundo

**Affiliations:** RMNCH Unit, Global Health Programs, FHI 360 359 Blackwell Street, Durham, NC 27701 USA; Averting Maternal Death & Disability, Mailman School of Public Health, Columbia University, New York, NY USA; Global Health Fellows Program II, United States Agency for International Development (USAID), Washington, DC USA; MEASURE Evaluation/Carolina Population Center, University of North Carolina at Chapel Hill, Chapel Hill, NC USA; Department of Maternal and Child Health, Gillings School of Global Public Health, University of North Carolina at Chapel Hill, Chapel Hill, NC USA; MCSP/Jhpiego, Maputo, Mozambique; Departamento de Saúde da Comunidade, Faculdade de Medicina, Universidade Eduardo Mondlane, Maputo, Mozambique

**Keywords:** Maternal mortality, Direct and indirect causes of death, Mozambique, Malaria, HIV infection

## Abstract

**Background:**

The paper’s primary purpose is to determine changes in magnitude and causes of institutional maternal mortality in Mozambique. We also describe shifts in the location of institutional deaths and changes in availability of prevention and treatment measures for malaria and HIV infection.

**Methods:**

Two national cross-sectional assessments of health facilities with childbirth services were conducted in 2007 and 2012. Each collected retrospective data on deliveries and maternal deaths and their causes. In 2007, 2,199 cases of maternal deaths were documented over a 12 month period; in 2012, 459 cases were identified over a three month period. In 2007, data collection also included reviews of maternal deaths when records were available (*n* = 712).

**Results:**

Institutional maternal mortality declined from 541 to 284/100,000 births from 2007 to 2012. The rate of decline among women dying of direct causes was 66 % compared to 26 % among women dying of indirect causes. Cause-specific mortality ratios fell for all direct causes. Patterns among indirect causes were less conclusive given differences in cause-of-death recording. In absolute numbers, the combination of antepartum and postpartum hemorrhage was the leading direct cause of death each year and HIV and malaria the main non-obstetric causes. Based on maternal death reviews, evidence of HIV infection, malaria or anemia was found in more than 40 % of maternal deaths due to abortion, ectopic pregnancy and sepsis. Almost half (49 %) of all institutional maternal deaths took place in the largest hospitals in 2007 while in 2012, only 24 % occurred in these hospitals. The availability of antiretrovirals and antimalarials increased in all types of facilities, but increases were most dramatic in health centers.

**Conclusions:**

The rate at which women died of direct causes in Mozambique’s health facilities appears to have declined significantly. Despite a clear improvement in access to antiretrovirals and antimalarials, especially at lower levels of health care, malaria, HIV, and anemia continue to exact a heavy toll on child-bearing women. Going forward, efforts to end preventable maternal and newborn deaths must maximize the use of antenatal care that includes integrated preventive/treatment options for HIV infection, malaria and anemia.

## Background

The east African country of Mozambique fits a paradigm of obstetric transition where maternal mortality and fertility remain high, and access to care and health system infrastructure continue to be a challenge. These characteristics define Stage 2 of the obstetric transition, where direct causes of maternal mortality predominate but a sizeable number of deaths are linked to infectious diseases [1]. It is also a country where the demand for maternity services is well-established and increasing [2].

Indirect maternal deaths are linked to previously existing diseases that are aggravated during pregnancy, or to diseases developed during pregnancy but are not due to direct obstetric causes [3]. They account for 27.5 % of maternal deaths worldwide according to the most recent systematic review for 2003–09; the percentage in sub-Saharan Africa is 28.6 % [4]. Both the first systematic review (1998–2002) and the 2003–2009 review highlighted the importance of indirect deaths in sub-Saharan Africa [5].

Women of reproductive age in Mozambique face a sobering circle of risk when they become pregnant—an increased risk of malaria, and of HIV infection and transmission. If a woman is HIV-infected, she is at increased risk of malaria complications, including severe anemia and maternal death, and complications for the child. These risks are in addition to the well-known obstetric risks faced by every woman who becomes pregnant [6]. HIV-infection in pregnancy increases the risk of maternal death by about eight times as well as the odds of developing certain obstetric complications such as intrauterine infections [7–9]. Infectious diseases can be prevented or treated and anemia can be reduced if not eliminated when women attend antenatal services able to address these problems. Antenatal care also provides the opportunity to detect and treat hypertensive diseases and infections, while a skilled professional at birth and emergency medical back up services should address the obstetric threats.

According to the 2013 estimates from WHO, UNICEF, UNFPA and the World Bank, Mozambique is “making progress” towards achieving the Millennium Development Goal (MDG) for improving maternal health; the maternal mortality ratio (MMR) currently stands at 480/100,000 live births. Between 1990 and 2013, the average annual decline in the MMR was 4.3 %, amounting to a 64 % decline since 1990, only 11 percentage points shy of achieving the 75 % reduction called for by the MDG 5 [3]. The percentage of facility births has increased from 47 % to 55 % according to the 2003 and 2011 Mozambique Demographic and Health Surveys [10, 11].

Sub-Saharan Africa accounts for 62 % of global maternal deaths as well as 91 % of the maternal deaths attributed to HIV/AIDS [3]. The contribution of HIV/AIDS to pregnancy-related mortality in this region has been estimated to be as much as 50 %, where the prevalence of HIV is 15 % or higher, and as low as 3 % [8, 12]. Measurement of attribution is clouded with concerns over the lack of standard approaches and when HIV should be considered an indirect cause or a coincidental cause [12].

The Mozambique 2007–2008 Post-Census Mortality Verbal Autopsy (INCAM) study estimated that HIV was the second leading cause of death in the general population [13]. Based on these data Singh et al. estimated that 18 % of all maternal deaths were caused by HIV and another 23 % by malaria [14]. The newest maternal mortality trends from WHO, UNICEF, UNFPA and the World Bank estimate that 13 % of maternal deaths in Mozambique are attributable to AIDS-related indirect maternal causes [3]. In an autopsy study of 139 maternal deaths in Mozambique’s capital, Menéndez and coauthors reported that non-obstetric conditions accounted for 56 % of deaths. In decreasing order of importance were HIV/AIDS, bronchopneumonia, severe malaria and meningitis. The primary obstetric cause of death was hemorrhage. They also observed that the majority of HIV/AIDS related maternal deaths were dying of underlying causes such as tuberculosis and Kaposi’s sarcoma [15]. In a second paper using the same autopsy dataset Ordi and coauthors found discrepancies between clinical diagnoses and autopsy results in 40 % of maternal deaths. They found not only a high rate of false negative diagnoses for infectious diseases but also a high false positive rate for eclampsia [16]. Where autopsies and diagnostic tests are not routine, misclassification is a common problem undermining the accuracy of causes of maternal deaths [3]. Frequent misclassification errors include epilepsy and eclampsia, cerebral malaria and eclampsia, malaria and other febrile illnesses, and those that surround abortion where deaths may be classified as hemorrhage or sepsis [4, 17].

Malaria is endemic throughout the country and transmission occurs throughout the year. It is the primary cause of death in Mozambique and its impact on the health system is heavy with 44 % of outpatient visits due to malaria [14, 18]. Its negative impact on pregnancy outcomes is well known, including maternal anemia, low birth weight, fetal growth retardation, pre-term delivery, spontaneous abortion, stillbirth, and neonatal death [19, 20]. The 2007 National Malaria Indicator Survey observed that 18 % of pregnant women were positive for malaria parasites and parasite density decreased as parity increased, confirming a higher risk status (less immunity) among women pregnant for the first time [18]. In a large referral hospital in the capital of Maputo, researchers found a significant relationship between seasonality, maternal mortality and the role of malaria. For three consecutive years (2001, 2002 and 2003), malaria was the primary cause of maternal mortality at this hospital [21].

Population-based national data on cause of maternal death are lacking in most of sub-Saharan Africa and elsewhere where the burden of maternal mortality is high. Yet one of the principles guiding program planning toward ending preventable maternal mortality is for every country to understand the primary causes of maternal death. Only then can the most appropriate interventions be identified and prioritized [22]. Available data tend to be based on subnational areas or single facility data and commonly reflect institutional maternal deaths only. Although the full demographic picture is missing, as the percentage of births in facilities increases, facility-based data will play an increasingly valuable role in understanding the causes of maternal deaths.

The objectives of this descriptive paper are to describe changes between 2007 and 2012 in the magnitude of institutional maternal mortality, the causes of institutional maternal deaths, and to highlight the extent to which HIV, malaria and anemia contribute to maternal mortality. Finally, we describe changes in the level of facility where maternal deaths occurred and the public health system’s availability of treatment for HIV infected pregnant women as well as treatment and prevention of episodes of malaria.

## Methods

Two similar national cross-sectional surveys of health care facilities conducted five years apart are the data sources for this secondary data analysis. Table 1 summarizes key aspects of the two surveys: the *Needs Assessment in Maternal and Neonatal Health in Mozambique, 2007–2008* and the *Needs Assessment for Emergency Obstetric and Neonatal Care in Mozambique, 2012.* Further information about the methodology of the surveys can be found in the final reports [2, 23].

**Table 1:** Differences and similarities between the 2007 and 2012 national health facility assessments

	2007–08	2012
Scope	National	National
Sampling	All hospitals and health centers in district capitals; 40 % stratified systematic random sample of lower level health centers and posts; sampling frame restricted to facilities that had attended at least one delivery in the last year *n* = 450	Census of all facilities that had attended at least one delivery in the last year *n* = 946
Weighting	Data required probability weighting to account for sampling at lower level health centers and posts and adjusted for non-response	Self-weighting
Data collectors	Range of health professionals	Medical students from the University of Eduardo Mondlane
Questionnaires	Paper draws from 3 of 17 questionnaires: 1) summary of service statistics for each facility, 2) maternal death reviews, 3) inventory of infrastructure, equipment, supplies and drugs for maternal and neonatal health	Paper draws from 2 of 6 questionnaires (similar to those used in 2007–08): 1) summary of service statistics, 2) inventory of infrastructure, equipment, supplies and drugs. These were adapted from AMDD tools [30]. Maternal death reviews were not administered.
Primary sources of data	Information on maternal deaths was collected from maternity, postpartum and gynecology logbooks, operating theatre, discharge and morgue registers, maternal death reviews/audits. Data collectors also asked staff for other facility-specific sources of information on maternal deaths. Information on institutional deliveries was collected from the relevant subset of registers. The use of monthly summaries of events (births or deaths) was discouraged due to concerns for accuracy.	Same as 2007–08
Service statistics reference period and number of events	12 months (November 2006—October 2007). The number of unweighted maternal deaths was 2,199, unweighted institutional deliveries = 312,537.	3 months (August-October 2012). Number of maternal deaths = 459, institutional deliveries = 161,986.
Overall data collection period	November 2007-January 2008	November-December 2012
Mozambique IRB approval and privacy safeguards	National Committee for Bioethics in Health IRB 00002657, Ministry of Health of Mozambique. All patient data were de-identified and analyzed in the aggregate; no names were collected.	Department of Reproductive, Child and Adolescent Health, within the Mozambique Ministry of Health’s National Directorate for the Promotion of Health and Disease Control. Like 2007, no names or identifying characteristics of patients were collected.

Both surveys were designed to provide the Ministry of Health and its partners with information for planning and strategic decision-making; to assess progress on the reduction of maternal mortality using indicators for monitoring emergency obstetric care [24]; to provide inputs for implementing interventions to reduce early neonatal mortality, prevent unwanted pregnancies, and to better understand the relationship between the availability of resources, quality of care and their impact on maternal newborn morbidity and mortality. The 2007 assessment was designed to collect information on a more ambitious range of topics, including investigating facility capacity to repair obstetric fistula, availability of maternity waiting homes, and interviews with service users. The 2012 health facility survey had twice the sample size but a more limited substantive scope.

In both surveys the causes of maternal deaths that were collected as part of the summary of service statistics were defined by the parameters set out by WHO in the ICD-10 that allows for direct and indirect causes of maternal death [3]. Some variation in the cause-of-death options occurred across the two surveys. In 2007 all deaths were categorized as falling into one of the following underlying causes of death: antepartum or postpartum hemorrhage, prolonged or obstructed labor, uterine rupture, postpartum sepsis, severe pre-eclampsia, eclampsia, abortion complications, ectopic pregnancy, other direct causes, HIV/AIDS, malaria and other indirect causes. The questionnaire did not allow for undetermined causes of death; it is likely that they were assigned to either the “other direct” or “other indirect” categories.

In 2012 the causes of death were virtually the same except that retained placenta was added to the questionnaire (and in this analysis retained placenta was combined with postpartum hemorrhage), and indirect causes were broken down into the following mutually exclusive categories: malaria, severe anemia, malaria + severe anemia, HIV/AIDS, HIV/AIDS + malaria, HIV/AIDS + anemia, other indirect causes, reflecting the growing awareness of the high rates of co-infection and co-morbidities. Finally, a category for unknown or unspecified causes of death was added.

In both surveys, clinical definitions were covered during the training of data collectors, all of whom had a medical or public health background. Definitions of severe obstetric complications could be found in the 2012 data collectors’ manual but were not included in the 2007 manual. Data collectors were expected to extract the cause of death from the registers and logbooks found in the facility. Diagnostic test results were not a prerequisite for assigning cause of death, and we do not know how many reported diagnoses were confirmed with diagnostic tests. No systematic physician review of these causes of death took place in 2007 or 2012.

In addition to the service statistics, a maternal death review (MDR) was a second source of information on maternal deaths available only for 2007. It collected demographic characteristics, obstetric history, and details related to each woman’s clinical condition and medical treatment. This module was completed for 712 of the 2,199 women identified in the summary of service statistics. An MDR was completed for every maternal death whose clinical records and files could be located (see Table 3). Patient records were supplemented by death reports performed by the Maternal Death Audit Committee, if the cases were audited, and if case notes and reports were available. At the end of the questionnaire, the data collector was given the opportunity to reassign a cause of death *only if their conclusion differed from that which was found in the patient’s records*. All MDRs were further reviewed by a team of physicians before data entry; the final cause appears in Table 3. No attempt was made to reconcile the cause of death as it appeared in the MDR with that in the summary of service statistics (see Table 2).

**Table 2:** Number of weighted and unweighted institutional maternal deaths and overall and cause-specific institutional maternal mortality ratios, by year

	Deaths reported in 2007 (12 months of data)					Deaths reported in 2012 (3 months of data)			
	n (unwt)	n (wt)	MMR per 100,000 births^a^	95 % Confidence Interval		n (unwt)	MMR per 100,000 births^a^	95 % Confidence Interval	
Overall institutional MMR	2,199	2,596	541	364.7	718.3	459	284	168.6	400.0
Direct causes of death	1,450	1,689	352	231.0	473.1	192	119	63.5	174.4
Antepartum hemorrhage	113	133	28	15.8	40.3	19	12	4.8	18.8
Postpartum hemorrhage/at delivery	129	183	37	23.5	51.2	52	32	11.5	52.9
Severe pre-eclampsia/eclampsia	230	246	52	15.7	87.3	29	18	8.8	27.2
Prolonged/obstructed labor	158	212	44	22.4	66.4	21	13	−0.8	26.8
Uterine rupture	130	139	29	18.5	40.0	22	14	6.1	21.1
Sepsis	101	107	23	12.3	33.0	28	17	7.3	27.4
Ectopic pregnancy	86	86	18	−7.1	43.6	9	6	−1.1	12.3
Abortion	104	106	22	9.7	35.2	7	4	−2.0	10.6
Other direct causes^b^	399	476	113	58.5	166.6	5	3	0.0	6.2
Indirect causes	749	906	189	83.6	295.3	226	140	50.9	229.1
Malaria	196	240	56	1.9	109.7	63	39	−3.0	81.1
Malaria + anemia	-	-				27	17	−2.6	36.0
HIV	197	246	57	24.5	89.8	57	35	−11.4	82.0
HIV + anemia	-	-				8	5	−1.2	11.1
Malaria + HIV	-	-				16	10	−6.3	26.1
Anemia	-	-				34	21	9.8	32.3
Other indirect causes^c^	356	420	98	32.3	163.4	21	13	3.0	23.0
Unspecified/unknown^d^						41	25	2.6	48.2

^a^2007 unweighted number of institutional deliveries = 312,537, weighted = 478,308. 2012 required no weighting, number of institutional deliveries = 161,986

^b^In 2012 other direct causes included suicide (rat poisoning), polyhydramnios, pyomyosites, complications from anesthesia or surgery

^c^In 2012 other indirect causes included intoxication with tradtional medicines, asthma, pulmonary edema, respiratory insufficiency, meningitis, meningoencephalitis, choked, brain tumor, hepatitis, cardiovascular disease, kidney failure

^d^In 2007 unspecified or unknown causes of death were grouped with “other” direct or indirect causes.

wt = weighted; unwt = unweighted

During the secondary data analysis, several questions in the review were used to determine if any evidence existed of HIV infection, malaria or anemia, and whether one or more played a role in the woman’s condition. If one or more of these questions below indicated that a woman was HIV infected, had a diagnosis of malaria or anemia, she was coded accordingly. Responses were pre-coded but an optional “other” category existed and these “other” responses were re-coded. The review questions were:

1. If the woman was referred, what were the reasons for that referral?
2. Diagnosis on admission
3. After your review of the woman’s charts and logbook data, what was the direct/indirect cause of death?
4. If the woman suffered from an illness or medical condition prior to the pregnancy, what was that illness or condition?

The MDR data were not weighted since almost no reviewed deaths occurred at the low level health centers and posts. The health facility itself was the unit of analysis for the assessment of the availability of infrastructure, equipment and drugs.

This paper presents a descriptive comparison of the 2007 and 2012 data. Secondary analysis was performed using Stata version 13 and SPSS version 17. For the 2007 data, we show weighted and unweighted numbers, but rates or percentages are based on weighted data, with the exception of the MDR data as stated above. Weights were calculated as the inverse of the probability of selection and adjusted by facility non-response. The 95 % confidence intervals are shown for the institutional MMR estimates in Table 2; all figures for Table 2 were calculated within Stata and took into account the weighting and provincial stratification. Although some of the confidence intervals include a negative integer, for practical purposes the lower bound was zero. These intervals should be considered informal testing as the two datasets were not merged. The institutional mortality ratios use as their numerator only deaths that occurred after admission to the facilities surveyed, and the denominator is based on the number of deliveries taking place in the same facilities over the same 12 or three month time period, depending on the survey.

The protocols for the primary data collection were approved (see Table 1) by local entities but the current secondary data analysis did not meet the regulatory definition of research with human subjects per the U.S. Code of Federal Regulations Title 45, Part 46.102(f), since the dataset obtained was completely de-identified. Similarly, the use of informed consent was deemed unnecessary for the secondary data analysis, however, permission to use the data from the two surveys was granted by the Ministry of Health’s National Directorate for the Promotion of Health and Disease Control, by UNFPA/Mozambique (a major funder of both surveys) and the University of Eduardo Mondlane, the assessment implementing partner in 2012.

## Results

### Magnitude of maternal mortality and changes in causes of maternal death

Based on the summaries of service statistics for each assessment, the institutional MMR declined by 47 % between 2007 and 2012, from 541/100,000 births to 284/100,000 (Table 2). In 2007, direct deaths were reported to have been associated with 352 maternal deaths per 100,000 births versus 119 in 2012, a statistically significant decline of 66 % (the two sets of 95 % confidence intervals do not overlap). The decline among indirect causes of maternal death was only 26 %, and not significant. Cause-specific institutional MMRs declined by more than 50 % for all direct causes except for postpartum hemorrhage and sepsis, and again, none was significant. The categorization of specific indirect causes between 2007 and 2012 changed, allowing combinations of causes to be reported in 2012, and making comparisons between the two surveys difficult. For example, if we combined maternal deaths due to malaria alone, malaria + anemia, and eight of the 16 malaria + HIV deaths (assuming we evenly split the 16 malaria + HIV deaths between malaria and HIV), and compared this ratio with that of the single diagnosis of malaria in 2007, the MMR for malaria changes from 56 to 60/100,000 deliveries. A similar exercise for HIV-related deaths produces a decline from 57 to 45/100,000. Another challenge is the large number of maternal deaths that appear as “other” direct and indirect causes in 2007. If these two groups were reassigned to “unspecified causes” then the decline in the direct institutional MMR would have been 53 % (instead of 66 %) and the 26 % decline in the indirect MMR becomes an increase of 37 %. However, the original categories are maintained rather than undermine the integrity of the data, assuming that allocation to one category over another was not arbitrary.

The primary obstetric cause of death in both years was hemorrhage, combining antepartum and postpartum hemorrhages (12 % of all deaths in 2007 and 15 % in 2012). In 2007, 10 % of all maternal deaths were attributed to HIV/AIDS and 9 % to malaria. In 2012, because the categorization allowed multiple conditions (malaria + anemia, HIV + anemia, malaria + HIV), the comparison is not possible without double-counting.

### Changes in location of deaths and births

Where women died in the hierarchy of the health system changed over the five year interval: in 2007, 49 % of maternal deaths took place in the largest urban hospitals (central, general and provincial hospitals), 16 % in rural/district hospitals, and 34 % in health centers and posts. In 2012, this distribution shifted to 24 % of deaths taking place in the largest urban hospitals and 41 % in rural/district hospitals. The percentage of deaths occurring in health centers/posts remained virtually the same (data not shown). This shift took place after 15 health centers were upgraded to the status of rural hospitals in 2012. No shift in where births took place was observed: in 2007, 15 % of births took place in the largest urban hospitals, 11 % in rural hospitals and 74 % in health centers and posts. The distribution in 2012 was 13 %, 15 % and 72 %, respectively (data not shown).

### Maternal death reviews and comorbidities

The 712 maternal death reviews, a subset (32 %) of the 2,199 deaths that occurred in 2007, allowed us to study in detail selected characteristics of the women who did not survive. A comparison of the two distributions of causes of death (among all reported maternal deaths and all deaths reviewed) suggests a larger proportion of direct deaths (74 %) among the reviewed deaths than all reported deaths (65 %). The MDRs reflect few “other” direct or indirect causes and has a category for undetermined causes. These aspects and the physician review in assessing and assigning causes that was part of the MDR process suggest a higher quality of reporting. However, the 712 reviewed deaths may not be representative of the 2,199 total deaths. More than half (54 %) of the MDRs were drawn from the most urban hospitals (central, general or provincial hospitals), 28 % from rural hospitals and 18 % from health centers (data not shown). Comparing this distribution to the 2,199 maternal deaths, the MDRs under-represent the deaths occurring at health centers/posts. This finding might reflect better record keeping and more systematic death reviews or audits at higher level facilities than at health centers and posts.

The differences between distributions, however, do not diminish the objective of the analysis intended with this table. Table 3 shows to what extent malaria, anemia, HIV infection or any one of these three morbidities contributed to the woman’s condition at death, for each reported cause of death. The existence of these co-morbidities was determined by the four questions found in the review and described in the methods section. Among women who died of direct causes, malaria contributed to 13 % of cases, anemia to 13 %, and HIV to 11 %, and evidence of any one of the three co-morbidities was present for 30 % of the direct causes. These three categories were not mutually exclusive, in other words, a woman who died of antepartum hemorrhage could have been both HIV+ and anemic.

**Table 3:** Distribution of 2007 causes of death according to data source and percent of maternal death reviews for which malaria, anemia, HIV infection, or any one of the three morbidities contributed to the woman’s condition

	Maternal deaths reported in service statistics		Maternal deaths reviewed		Malaria	Anemia	HIV	Any 1 of 3
	n	% (wt)	n	%	%	%	%	%
Total	2,199	100	712	100	21	14	22	44
Direct causes	1,450	65	528	74	13	13	11	30
Antepartum hemorrhage	113	5	37	5	5	19	14	30
Postpartum hemorrhage	129	7	101	14	7	13	10	25
Severe pre-eclampsia/eclampsia	230	10	91	13	23	3	13	33
Prolonged/obstructed labor	158	8	23	13	17	0	4	22
Uterine rupture	130	5	116	16	3	9	2	13
Sepsis	101	4	80	11	16	19	21	41
Ectopic pregnancy	86	3	21	3	14	38	0	43
Abortion	104	4	50	7	26	24	26	56
Other direct causes	399	19	9	1	11	11	0	22
Indirect causes	749	35	173	24	49	19	56	89
Malaria	196	9	52	7	100	14	10	100
HIV	197	10	76	11	24	15	100	100
HIV + Malaria	-	-	10	1	100	10	100	100
Anemia	-	-	13	2	15	100	31	100
Embolism	-	-	7	1	0	14	0	14
Other indirect causes	356	17	15	2	13	0	13	13
Causes unknown			11	2	0	9	0	9

Several specific direct causes of death stand out as having a large percentage of co-morbidity: 21 % of deaths due to puerperal sepsis and 26 % of abortion cases had evidence of HIV infection; 23 % of severe pre-eclampsia/eclampsia cases and 26 % of abortion deaths registered evidence of malaria; while anemia was implicated in 38 % of the deaths due to ectopic pregnancy and 24 % of abortion deaths. Evidence of HIV infection, malaria and/or anemia was found among 56 % of the abortion deaths.

Evidence of HIV infection, malaria and anemia were, by definition, more prevalent among women who died of indirect deaths: evidence of HIV infection was documented among 56 % of the 173 women who died of indirect causes, malaria among 49 %, anemia among 19 %, and one or more of the three co-morbidities was found in 89 % of the cases. Among the 52 reported malaria deaths, HIV infection contributed to 10 % and anemia to 14 %.

### Mapping prevalence of HIV infection and malaria and institutional maternal deaths where these infections contributed

**Fig. 1:**
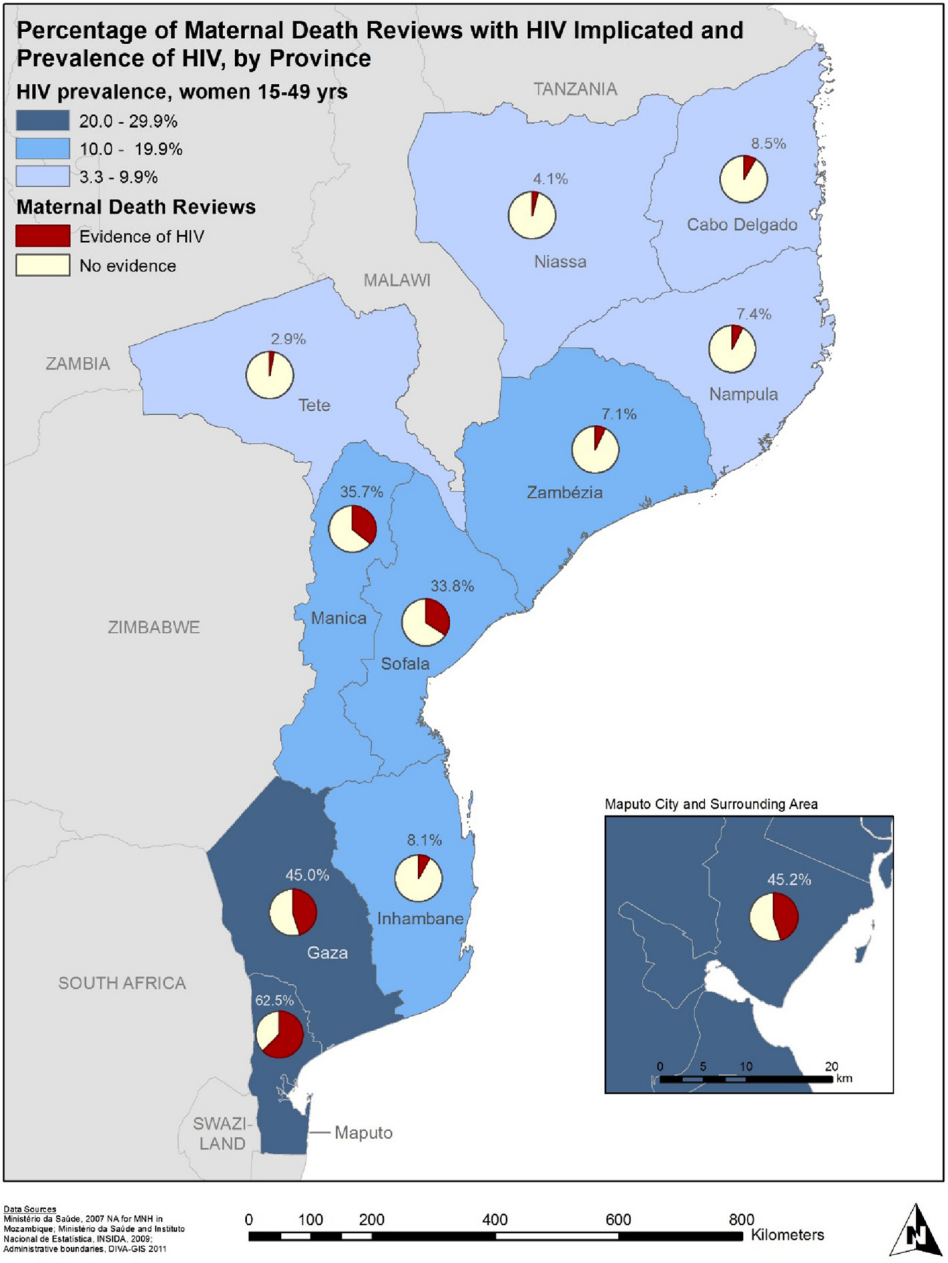
Map of maternal death reviews, with HIV implicated, 2007

The percentage of MDRs with evidence of HIV infection reported in each province mirrored the prevalence of HIV infection among women ages 15–49 overall: lower percentages in the north where the prevalence of HIV has been the lowest and higher percentages in the south, Zambézia being the exception (Fig. 1). Despite an HIV prevalence of 15 % in Zambézia, only 7 %, or 5 of 70 maternal death reviews reported HIV infection [25].

**Fig. 2:**
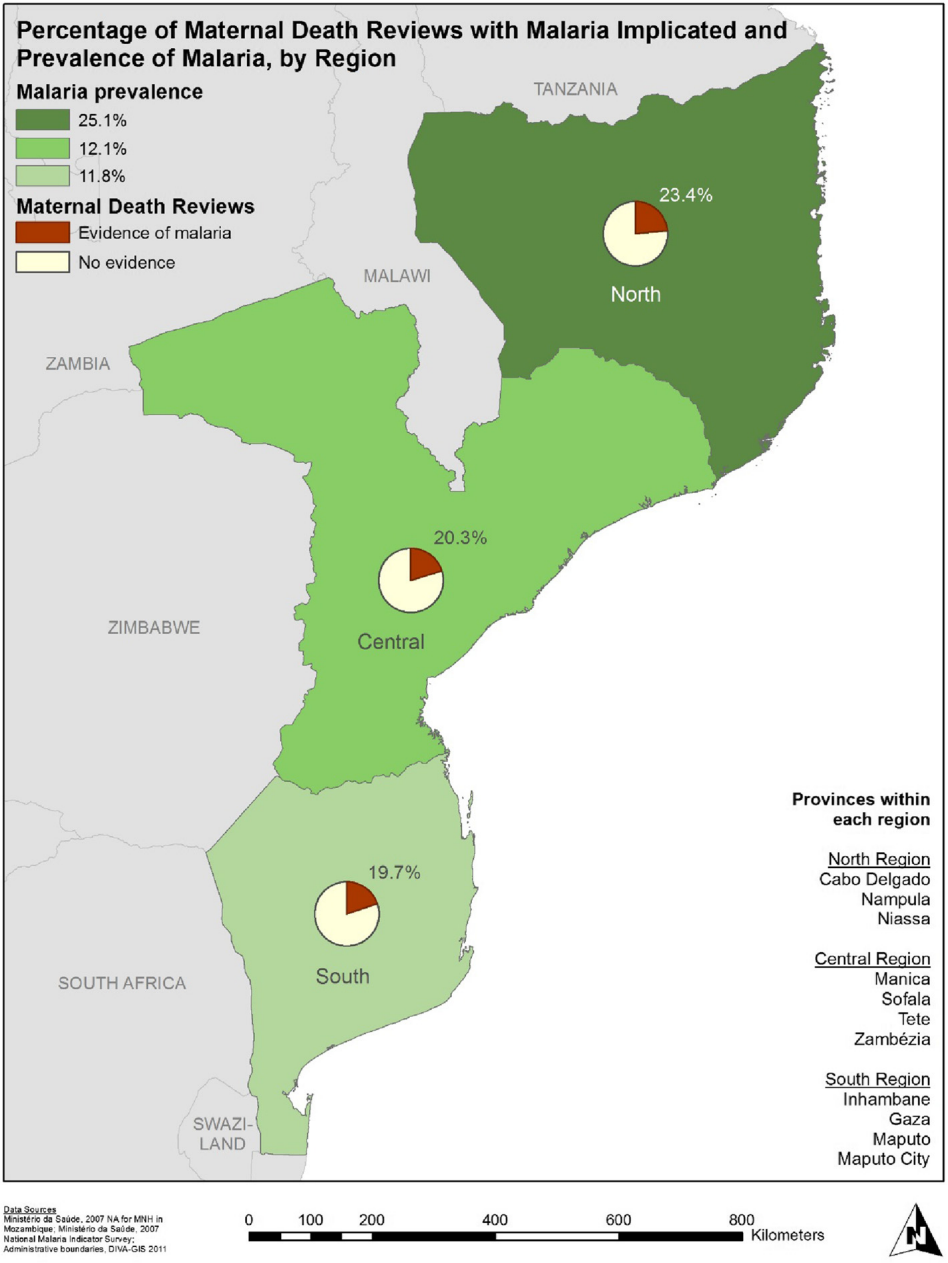
Map of maternal death reviews, with malaria implicated, 2007

A reversed geographical pattern is seen with malaria (Fig. 2), where the northern region had the highest prevalence of malaria among pregnant women (25 %) and the highest percentage of case reviews with indications of malaria (23 %). The south had the lowest malaria prevalence (11.8 %) and also registered the smallest percentage of maternal deaths where malaria was implicated (19.7 %), though differences between the central and southern regions were small [18].

### Availability of treatment for HIV/AIDS and malaria

The two surveys did not focus on supplies used during antenatal care so an assessment of the availability of important preventive measures such as insecticide treated bednets (ITNs) or iron folate was limited. Nevertheless, we were able to measure the percentage of health facilities by type of facility that had certain antiretrovirals and antimalarials in stock at the time of data collection (Table 4). For all like items, greater availability at each level of facility was observed in 2012 compared with 2007, but the increased availability at the health center/post level was particularly dramatic. Additional drugs in each category were captured as well, but they differed between surveys.

**Table 4:** Percentage of facilities with drugs in stock for HIV and malaria, by type of facility and year

	Central, Provincial & General Hospitals		Rural & District Hospitals		Health Centers & Posts		Total	
	2007	2012	2007	2012	2007	2012	2007	2012
	*n* = 16	*n* = 14	*n* = 26	*n* = 41^a^	*n* = 347	*n* = 891	*n* = 389	*n* = 946
	% (wt)	%	% (wt)	%	% (wt)	%	% (wt)	%
Antiretrovirals								
Nevirapine	71	93	74	93	23	88	26	88
AZT	65	100	74	83	20	81	23	81
3TC + AZT/D4T	71	na	73	na	18	na	21	na
3TC	na	86	na	73	na	59	na	60
Antimalarials								
Artemether + Lumefantrine (Coartem)	81	86	87	90	18	86	22	86
Quinine (inj)	74	100	54	85	22	70	24	71
Quinine (oral)	70	100	47	88	12	63	14	64
Artesunate	na	29	na	5	na	7	na	7
SP (IPTp)	94	na	83	na	32	na	35	na

*AZT* zidovudine, *3TC* lamivudine, *D4T* stavudine, *SP* sulfadoxine-pyrimethamine (Fansidar), *IPTp* intermittent presumptive treatment during pregnancy, *na* not applicable, *inj* injection, *wt* weighted

^a^15 health centers in 2007 were reclassified as rural hospitals by 2012

## Discussion

This paper triangulates three national datasets to describe the magnitude and causes of maternal mortality, two datasets reflecting two points in time (2,199 and 459 maternal deaths in 2007 and 2012, respectively) that use a similar methodology, and a third dataset that consisted of 712 maternal death reviews, a subset of the 2,199 maternal deaths identified in the 2007 MNH assessment. These datasets may contain the most comprehensive information available to shed light on the evolution of maternal mortality over the last decade.

During this five-year period, the findings of these two sequential health facility assessments suggest that the institutional MMR has declined by almost half, though technically speaking, the 95 % confidence intervals do overlap. The profile of institutional maternal mortality may also be changing to one where proportionally fewer maternal deaths are caused by obstetric complications and a greater proportion is attributable to non-obstetric causes. The declines among obstetric deaths were particularly noteworthy during the five-year interval. Data collection methods preclude us from making similar conclusions about declines among indirect causes of death. Meanwhile the MDRs revealed how frequently HIV infection, malaria and anemia were associated with women dying of obstetric causes. Health system efforts to reduce direct obstetric deaths may be succeeding as are efforts to increase institutional births, both of which are meaningful achievements.

This study complements the work by Singh et al*.* that drew from the 2007–08 INCAM study of deaths that occurred in 2006–07, a similar timeframe as the deaths in the 2007 MNH assessment [15]. Forty-three percent of the maternal deaths identified in the post-census study died within health care facilities [14], some of whom probably were counted in the 2007 MNH assessment. Table 5 summarizes the breakdown between direct causes of death and the primary non-obstetric causes for the different national data collection efforts. These sources point to the sizeable contribution that these two infectious diseases make on maternal mortality.

**Table 5:** Summary of causes of death by data source

Data Source	No. of maternal deaths	Direct causes	HIV-related	Malaria-related	Other indirect	Unknown
2007–08 INCAM	213	55 %	18 %	23 %	4 %	--
2007 MNH assessment	2,199	65 %	10 %	9 %	17 %	--
2007 MDRs	712	74 %	12%^a^	7 %	5 %	2 %
2012 EmONC assessment	459	42 %	17%^b^	20 %	12 %	9 %

^a^1 % of the MDR deaths were attributed to malaria + HIV and were included with HIV deaths

^b^3 % of 2012 deaths were attributed to malaria + HIV and are included with HIV deaths

Based on the 2007 MDRs and responses to ancillary questions, HIV infection, malaria and/or anemia contributed to 44 % of the maternal deaths, if not as primary causes, as co-morbidities. By focusing on the prevention and treatment of selected obstetric causes (postpartum hemorrhage, severe pre-eclampsia and eclampsia, and prolonged and obstructed labor/uterine rupture) as well as these three co-morbidities, the burden of maternal mortality in Mozambique could be significantly reduced. The integration of HIV and malaria services into reproductive health services, especially through the platform of antenatal care, has the scope for improving maternal and fetal/newborn outcomes. The shift towards more non-obstetric causes of death makes the target number and quality of antenatal visits all the more critical. It also raises cautionary concerns about overloading providers of antenatal care, the need for additional time and skills for different tests, educational messages, and the need for sufficient staffing, equipment and supplies.

### Program recommendations

HIV counseling, testing and antiretroviral therapy for HIV infected pregnant women is one such package of services, with strong evidence of the protective effect of antiretroviral therapy (ART) for HIV+ pregnant women, especially if they initiate therapy before pregnancy [26]. It is not clear how much effect ART is likely to have had in 2007 as the WHO guidelines for ART and improving maternal survival were not issued until 2010.

Where malaria transmission is stable and high, WHO has endorsed intermittent presumptive therapy (IPTp) with at least two doses of sulfadoxine-pyrimethamine (SP) during pregnancy and the use of insecticide-treated bednets for pregnant women [27]. Iron-folate supplementation for 90 days is also recommended. The Mozambican national policy for malaria in pregnancy stipulates three doses of SP and each is taken under observation during antenatal care. The 2011 Demographic Health Survey and the 2007 Malaria Indicator Survey indicate gaps in coverage and the quality of these services [11, 18]. Using mixed methods, Boene and colleagues looked at the acceptability of bednets and SP and found that bednets were the preventive intervention of choice, but they also found that women’s awareness of the adverse consequences of malaria in pregnancy especially for the fetus or newborn was low [28].

This paper also presents evidence of an increase in the availability of antimalarials and antiretrovirals, especially at the health center/post level, which should translate into greater access, utilization and protection. From the 2011 Mozambique Demographic and Health Survey, we know that 34 % of pregnant women slept under an insecticide treated bednet, 40 % of women with a live birth in the two years before the survey took antimalarials during the pregnancy, and 19 % received IPTp [11]. Given the finding in this study of no apparent decline in reported malaria deaths, it is possible that utilization of these protective measures had not increased sufficiently to have been measurable in 2012. Subsequent rounds of household surveys may shed light on the extent to which utilization has increased.

### HIV and direct obstetric complications

Some of our descriptive findings are in agreement with the literature on the relationship between HIV infection and the risk of direct obstetric complications. Two mechanisms of action may be at work: first, a biological increase in susceptibility due to immunosuppression but also social discrimination that could drive a delay in accessing facility-based care. Calvert and Ronsmans found that HIV infected pregnant women had more than three times the odds of developing puerperal sepsis compared with uninfected women [9]. Among the MDRs we also found a high percentage (21 %) of the 80 deaths due to sepsis were HIV-infected women while as many as 41 % reported signs of HIV, malaria, or anemia. Invasive non-typhoidal Salmonella infection is also associated with HIV, malaria and anemia, but often goes undiagnosed and may explain some of these findings. A similarly high proportion (43 %) of deaths due to ectopic pregnancy also reported signs of HIV, malaria, or anemia.

Among the MDRs were 50 deaths attributed to abortion and more than half also suffered from HIV infection, malaria, or anemia, all of which have a plausible biological mechanism of action. The data do not allow us to distinguish spontaneous pregnancy losses from attempts to terminate a pregnancy. Pressure to prevent pregnancies with contraceptive methods among HIV+ women has been observed in Mozambique [29]. The authors also found that the desire for pregnancy among HIV+ women was often strong. A healthy pregnancy in an HIV+ woman can be supported with IPTp, bednets, antimalarials, antiretrovirals, and iron folate for anemia.

### Limitations

Several limitations to this study should be noted. Some are challenges common to using secondary data designed for other purposes but also are common to studying causes of maternal mortality, especially at this scale – problems of sample size, validity as well as comparability. A certain amount of caution should be exercised when interpreting cause-specific changes over time. Assigning cause of death was dependent on accessible written records found in the facility and not necessarily driven by laboratory based diagnoses or autopsy reports, affecting both obstetric and non-obstetric causes. Comparability among indirect causes was hampered by changes in the data collection forms. The MDRs in 2007 may have more accurate diagnoses but we do not know how well they represent the deaths who were not reviewed. Health workers experience difficulties with coding and death certificate completion, and with the misclassification of maternal vs. non-maternal deaths, as well as specific causes. Identification of maternal deaths can be obstructed especially if there is no relationship of trust between unknown data collectors and health workers. Identifying indirect deaths is particularly difficult in these assessments since these women usually do not die in the maternity wing and the focus of the assessments was clearly on obstetrics and childbirth. Data collector fatigue is a common problem, data collectors may fail to request help due to concern about interrupting services and making demands on health workers, and they feel pressure to move on to the next facility. The careful review that might be expected at a single facility is difficult to sustain over hundreds of facilities. To minimize some of these sources of bias, data collectors were trained to be non-judgmental and to seek out multiple sources of information for facility deaths—to ask about facility specific sources, autopsy reports, mortuary logbooks, operating theatres, ob/gyn registries, and admission and discharge registries. Nevertheless, the categorization in 2007 of 19 % as “other direct” deaths and 17 % as “other indirect” deaths are indications of a lost opportunity to better inform programmatic efforts for maternal and newborn health. During this secondary data analysis the original 2007 questionnaires were not available, and these “other” deaths could not be explored. Women whose cause of death was unknown were likely to have been placed in these categories as were women with competing multiple causes, such as an obstetric and a non-obstetric cause of death. It is also highly likely that the “other” indirect deaths include cases of tuberculosis, hepatitis, pneumonia, cardiovascular disease, diabetes and other relatively common indirect causes.

Another concern for comparability derives from the different data collection periods. The 2007 assessment collected deaths over a period of 12 consecutive months. In 2012, the summary statistics were drawn from a period of only three consecutive months—August, September and October. We know that malaria-related deaths in Mozambique are seasonal, with peaks during and after the rainy season, which is October through March [21, 28]. According to Romagosa et al., the primary peak in Maputo is April, followed by a peak in October. In 2012, 106 maternal deaths were to women with signs of malaria and 58 % occurred in the month of October, suggesting that the data collection period included at least one of those peaks. However, it is possible that the three months of maternal deaths were not representative of those that would have occurred over a 12-month period.

### Methodological issues

For a complete understanding of the epidemiology of maternal death, the public health community aspires to population-based studies of the causes of maternal death, but as the percentage of births delivering in facilities increases, facility-based studies become more useful. In fact, the most recent systematic review of maternal deaths included facility-based distributions of causes of maternal deaths if at least 50 % of births were institutional [4]. The 2011 Mozambique Demographic and Health Survey found that 55 % of deliveries were conducted in facilities [11], while the 2012 EmONC assessment estimated an institutional delivery rate closer to 70 %, albeit using different methodologies [2]. Nevertheless, the institutional ratios can be helpful to program managers and clinicians in identification and prioritization of morbidities that health workers must be competent and equipped to treat or refer.

By measuring the magnitude of institutional maternal mortality with the maternal mortality ratio inevitably leads to a comparison between the institutional MMR and the population-based MMR. But they are not readily comparable given different measurement methodologies, sources of data, and the selection bias inherent among women who seek health care services in facilities. The 2013 MMR as measured by WHO, UNICEF, UNFPA, the World Bank and the United Nations Population Division was 480/100,000 births, substantially higher than the 2012 institutional MMR of 284. The higher figure is the result of multi-level regression modeling that takes into consideration an incomplete civil registration system and other types of data, and estimates separately the proportion of AIDS deaths that qualify as indirect deaths [3]. The 2012 institutional figure of 284 is likely to be low as we know that the three-month time period on which it was based failed to include the peak month of malaria deaths. Also, we can assume that maternal deaths were missed by the facility assessment. An important unknown is how many maternal deaths continue to take place outside health care facilities. According to the INCAM data of 2007, only 43 % of all maternal deaths occurred within health care facilities. Given the increase in institutional deliveries since 2007, it is possible that more deaths occur in facilities than in the community. The increase in institutional delivery is also likely to affect the patient profile, perhaps lowering the risk profile as more women experiencing a routine delivery seek services. This would translate into an increasing denominator for the institutional ratio. The next Mozambique Demographic and Health Survey, assuming it measures maternal mortality, will produce yet another population-based estimate that may confirm an MMR lower than the 2011 DHS estimate of 400.

In recognition of the growing importance of indirect causes of maternal mortality, the indicator “proportion of institutional maternal deaths due to indirect causes” was added in 2009 to a widely used set of indicators for monitoring emergency obstetric care and promoted by the UN agencies [24]. The rationale behind this indicator was first, to draw attention to the rising need for interventions in addition to emergency obstetric care, and second, to monitor the shift towards increasing indirect deaths as obstetric causes decline. However, as the research on the intersection between maternal mortality and HIV infection suggests, the term indirect may be less helpful than distinguishing between obstetric and non-obstetric causes or between deaths “with HIV” and deaths “from HIV.” The same could be said for malaria-related deaths.

## Conclusions

Mozambique’s pregnant women face multiple risks in an environment of high prevalence of HIV and malaria and where access to services can be a challenge. There are encouraging signs that institutional maternal mortality is decreasing, but infectious diseases are a larger proportion of the deaths. Moving forward, HIV and malaria prevention/treatment should be further integrated into maternal and child health platforms such as antenatal care, but with the necessary system support. In addition to looking at population level data sources when available, access to facility-based information on maternal causes of death is relatively easy and invaluable—and more countries should take advantage of this information readily at hand as well as implement maternal death reviews—as a means to prioritizing interventions needed to end preventable maternal and newborn deaths.
